# Antimicrobial resistance trends in bloodstream infections at a large teaching hospital in China: a 20-year surveillance study (1998-2017)

**DOI:** 10.1186/s13756-019-0545-z

**Published:** 2019-05-28

**Authors:** Lei Tian, Zhen Zhang, Ziyong Sun

**Affiliations:** 1 Department of Clinical Laboratory, Tongji Hospital, Tongji Medical College, Huazhong University of Science and Technology, Wuhan, Hubei Province China; 2 Department of Pharmacy, Tongji Hospital, Tongji Medical College, Huazhong University of Science and Technology, Wuhan, Hubei Province China

**Keywords:** Bloodstream infections, Bacterial pathogens, Antimicrobial resistance, Retrospective analysis, Patients, Central China

## Abstract

**Background:**

Bacterial bloodstream infections (BSIs) cause high morbidity and mortality worldwide in humans, but the pathogenic spectrum varies from region to region. Long-term monitoring of the pathogenic spectrum and changes in bacterial antibiotic resistance is hugely important for effective clinical therapy and infection control. This study examined the data for BSIs in Tongji Hospital, one of the largest teaching hospitals in China, in an attempt to gain better understanding of bacterial antibiotic resistance in China, focusing on central China.

**Methods:**

Data from Tongji Hospital for a 20-year period (1998–2017) were used for a retrospective analysis to understand the pathogenic spectrum of BSIs and the changes occurring in antimicrobial resistance in central China. The disk diffusion and E test methods were used for antimicrobial susceptibility testing according to Clinical & Laboratory Standards Institute methodologies, and the data were analyzed by WHONET 5.6 software.

**Results:**

The isolated pathogens mainly came from hospitalized patients not treated in intensive care units (ICUs), and accounted for 81.5% of the total (9130/11200). The most common Gram-negative and Gram-positive bacterial BSI-causing pathogens were *Escherichia coli* and *Staphylococcus aureus*, respectively. The detection rate for methicillin-resistant *S. aureus* (MRSA) in the hospitalized non-ICU patients increased from 8.4% in 1998–2002 to 63% in 2013–2017, while the detection rate for carbapenem-resistant (CR) *Klebsiella pneumoniae* was below 5% in 1998–2012 but increased to 34.9% in 2013–2017. In contrast, worryingly, the detection rate for CR *K. pneumoniae* in ICU patients increased from 0% in 2013 to 75% in 2016. *E. coli* displayed the highest sensitivity rates to imipenem, meropenem and amikacin, all of which were > 90%, followed by cefoxitin at > 80%, and cefoperazone/sulbactam at > 70%. *K. pneumoniae* isolates were most sensitive to imipenem, meropenem and amikacin antibiotics, with sensitivity rates exceeding 60%. *S. aureus* isolates were most sensitive to vancomycin, teicoplanin and trimethoprim/sulfamethoxazole, with sensitivity rates exceeding 90%.

**Conclusions:**

BSIs caused by CR *K. pneumoniae* clearly posed a severe challenge to infection control and treatment of ICU and non-ICU patients in this retrospective study, while MRSA was an issue for non-ICU patients.

## Background

Bloodstream infections (BSIs) inflict high morbidity and mortality on people from both developed and developing countries in the world. Analyzing the pathogenic spectrum of BSIs and the changes occurring in the antimicrobial resistance patterns of pathogenic bacteria from the data accumulated over many years is very important for clinicians and for infection control. However, the pathogenic spectrum and bacterial resistance patterns of BSIs reportedly differ in the different regions of the world. For example, in Malawi, Africa, the most common pathogens causing BSIs were found to be non-typhoidal salmonella, *Salmonella typhi*, and *Streptococcus pneumoniae* in an analysis of accumulated data over 19 years (1998–2016) [1]. In contrast, data from the European Antimicrobial Resistance Surveillance Network (EARS-Net, formerly EARSS, which includes 198 laboratories in 22 European countries) from 2002 to 2009 indicated that *Escherichia coli* and *Staphylococcus aureus* were the most common BSI-causing pathogens [2]. Similarly, the surveillance network data (Kor-GLASS) from South Korea in 2016–2017 showed that *E. coli* and *Staphylococcus aureus* were the most common BSI-causing pathogens [3], whereas the data for Japan showed that *E. coli*, *Staphylococcus aureus*, *Streptococcus* spp*.* and *Klebsiella* spp*.* were the most common BSI-causing pathogens [4].

The data for BSIs in China are very limited. Hence, in the present study, we analyzed the BSI data for Tongji Hospital in Wuhan, the capital city of Hubei Province in central China. Tongji Hospital is one of the largest Chinese teaching hospitals in China and from 2018 this hospital supplies three sub-districts with 6000 beds in total. Tongji Hospital was the first Asian hospital to pass the Kooperation für Transparenz und Qualiät im Gesundheitswesen certification in Germany and the Department of Laboratory Medicine was one of the first laboratories in China to pass both the International Organization for Standardization 15,189 and College of American Pathologists (CAP) certification in the USA. At the same time, Tongji Hospital also acted as the central network hospital for the Antimicrobial Resistance Surveillance System (HBRASS) in Hubei Province. The data from Tongji Hospital are, therefore, representative of the bacterial resistance patterns in central China. Our previous studies from HBRASS from 2014 to 2016 showed that the most common BSI-causing pathogens in Hubei Province were *E. coli, Staphylococcus aureus* and *K. pneumoniae* [5]. The data accumulated for Tongji Hospital over the last 20 years (1998–2017) is a very valuable and meaningful resource for understanding the pathogenic spectrum and bacterial resistance in BSIs in China, especially central China.

## Materials and methods

### Study design and procedures

This study was a retrospective analysis of all the data on blood cultures accumulated by the Microbiology Laboratory from the Department of Laboratory Medicine of Tongji Hospital from 1998 to 2017. Blood culture specimens from all patients including adults and children in Tongji Hospital were sent to the Microbiology Laboratory for testing. Blood culture was performed when the patient had the following clinical symptoms or signs [6]: 1. The patient’s body temperature was higher than 38 °C or lower than 36 °C, and was also the most common clinical situation for ordering blood culture. 2. The peripheral blood cell count showed an increase in white blood cells (WBCs) above 10 × 10^9^/L or a decrease in the WBC count below 4 × 10^9^/L. 3. A patient’s respiratory rate exceeded 20 breaths per minute or the arterial partial pressure of carbon dioxide was under 32 mmHg. 4. The patient’s heart rate exceeded 90 beats per minute. 5. The patient developed chills, coma, skin and mucosal bleeding, hypotension or multiple organ dysfunctionality. 6. The laboratory tests showed increases in inflammatory response indicators (e.g., C reactive protein, procalcitonin, and a positive G test, among others). Blood sample volumes were 8–10 ml per bottle for adult patients and 2–5 ml per bottle for children. For adult patients, each set included an aerobic bottle and an anaerobic bottle, but only an aerobic bottle was used for children. The instruments used for blood culturing were purchased from the BD Company (product models, 9120, 9240 and FX-400) and the BioMerier Company (3D instrument system). Samples from adults were analyzed on the BD and BioMerier systems; samples from children were analyzed on the BioMerier system.

The incubation period for common bacteria was 5 days, and 14 days for fungi. When it was suspected that the pathogen was a slow-growing bacterium like Brucella, the incubation period was extended appropriately. If a positive alarm occurred in the blood culture instrument, smear, stain, microscopic examination and transfection were carried out. When false positive results were found via blood smears, microscopic examination, or observing the growth curve from the instrument, the blood culture bottle would be returned to the instrument within one hour to continue the culture. Strain identification was carried out biochemically, using the automatic identification system (Vitek-2-compact, BioMerier Products) and/or the IVD-MALDI Biotyper (Bruker, Germany). Antimicrobial susceptibility tests were carried out according to Clinical & Laboratory Standards Institute (CLSI) approved procedures using disk diffusion and E test methods.

All laboratory operations were carried out in strict accordance with the standardized operating procedures of the hospital departments. The laboratories maintained good indoor quality control and external quality assessment was conducted. Before each batch of blood culture bottles was tested, quality control verification involved the use of standard strains (ATCC25923, 25922, 27852, 49619, and 49247). Monthly quality control verification of colorimetric GP and GN identification cards was carried out for the VITEK-2-COMPACT automatic identification instrument. ATCC8739, 25923, 90028 and a blank control were used for the quality control operations on the IVD-MALDI Biotyper for each batch. Antimicrobial susceptibility tests on the strains were carried out by the disk diffusion method and the E test method in accordance with CLSI guidelines. Antimicrobial sensitivity test paper was purchased from OXOID Company. ATCC 25922, 25923, 27853, 90028, 35218, 700603, 29213 were used for the quality control assessment of indoor antimicrobial sensitivity tests, which were performed weekly.

Since 1998, all the data including the patient’s age, sex, hospital department, hospital number, sample type, collection time, isolated pathogen(s) and drug sensitivity results have been stored in the WHONET system. Patients with incomplete data were not included in the analysis. Outpatient refers to patients with mild clinical symptoms. After a complete set of diagnostic and auxiliary examinations were conducted by the outpatient doctors, each patient was given a preliminary diagnosis. Outpatient doctors treated the patients symptomatically. When the outpatient doctors had doubts about a patient’s condition or the diagnosis was that of a serious and urgent case, the patient was admitted to the inpatient ward for further examination or surgical operation in the hospital. Outpatients included general outpatients and emergency patients. Inpatients were patients who were transferred to outpatient physicians in specialist wards for further treatment according to the type and severity of the disease.

### Statistical analysis

Interpretation of the antimicrobial susceptibility results was carried out in accordance with CLSI 2018 guidelines [7]. Antimicrobial sensitivity results were expressed in terms of sensitivity rates according to the CLSI M39 guidelines [8]. To avoid the effect of repetitive isolation of strains on antimicrobial sensitivity, the first isolation of strains from the same site of infection in a patient was analyzed according to CLSI M39 guidelines [8]. WHONET 5.6 software was used to analyze the antimicrobial susceptibility data. When the isolated strains were coagulase-negative staphylococcus, Corynebacterium, bacillus, Propionibacterium or other potential skin contaminants, they were considered as contaminants in this analysis. The clinical significance of a sample required two or more isolated blood cultures to confirm the diagnosis [9].

## Results

**Fig. 1:**
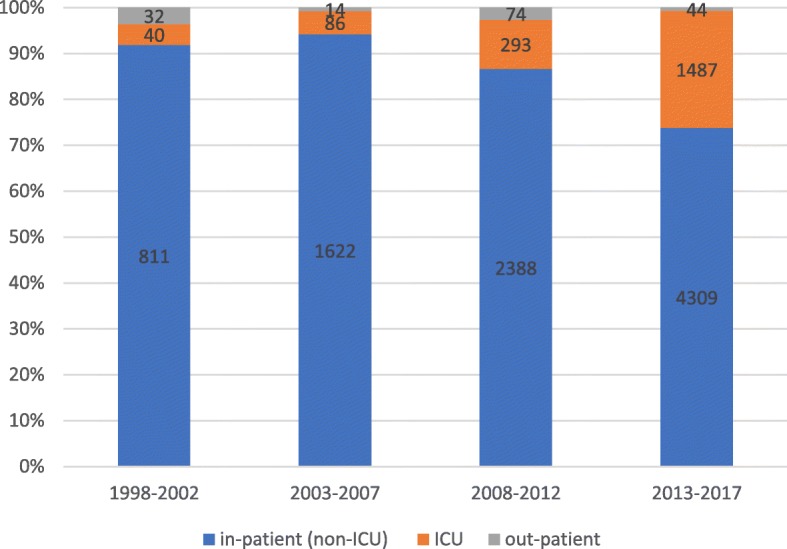
Distributions of pathogenic bacteria isolated from outpatients, ICU and non ICU patients in 1998–2017

**Fig. 2:**
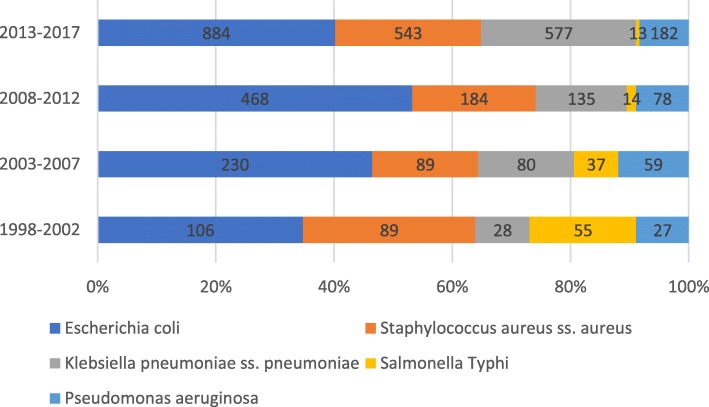
Distributions of the common pathogens of bloodstream infection in 1998–2017

From 1998 to 2017, 9130 non-intensive care unit (ICU) inpatients, 1906 ICU patients and 164 outpatient patients in Tongji Hospital had positive blood cultures. The proportion of hospitalized non-ICU patients with positive blood cultures was 81.5% (9130/11200) (Fig. 1). The most common pathogens detected were *E. coli* and *Staphylococcus aureus* from 1998 to 2017. *Salmonella typhi* ranked third in 1998–2002, and *K. pneumoniae* ranked third in 2003–2017 (Fig. 2). The limited number of strains isolated from outpatients and ICU patients meant that only the antimicrobial sensitivity results from the hospitalized non-ICU patients were analyzed.

**Fig. 3:**
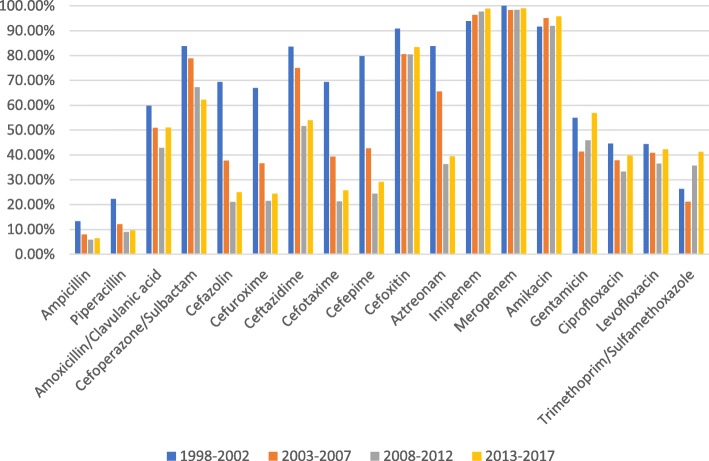
Sensitivity of *Escherichia coli* from non-ICU patients with BSI to commonly used antibiotics

The antimicrobial susceptibility data showed that the susceptibility rates for *E. coli* to imipenem, meropenem and amikacin exceeded 90%, while cefoxitin sensitivity ranged from 80 to 90%. In contrast, the susceptibility rates for ampicillin and piperacillin were almost below 20%. The sensitivity of *E. coli* to cefazolin, cefuroxime, cefotaxime, ceftazidime, cefepime and aztreonam showed a significant downward trend from 1998 to 2017, whereas quinolone sensitivity fluctuated slightly, and sensitivity to ciprofloxacin and levofloxacin ranged from 30 to 50% (Fig. 3).

**Fig. 4:**
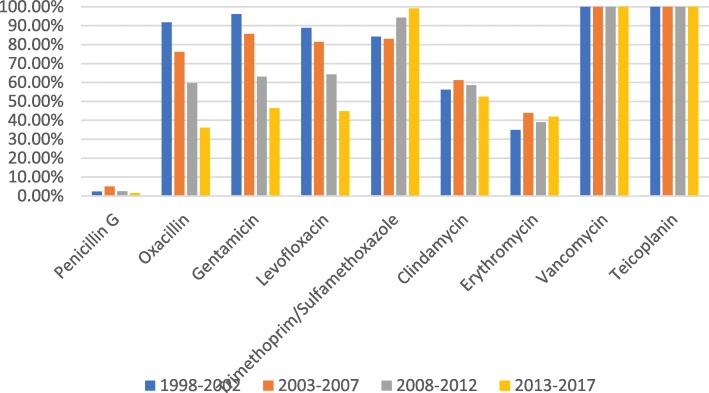
Sensitivity of *Staphylococcus aureus* to commonly used antibiotics in patients with BSI from non-ICU

**Fig. 5:**
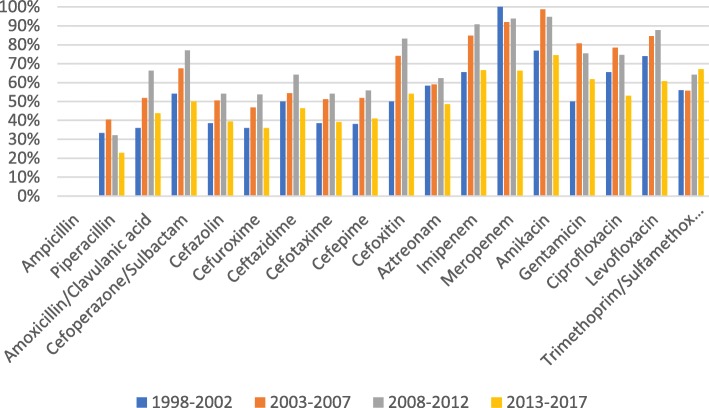
Sensitivity of *Klebsiella pneumoniae* from non-ICU patients with BSI to commonly used antibiotics

**Fig. 6:**
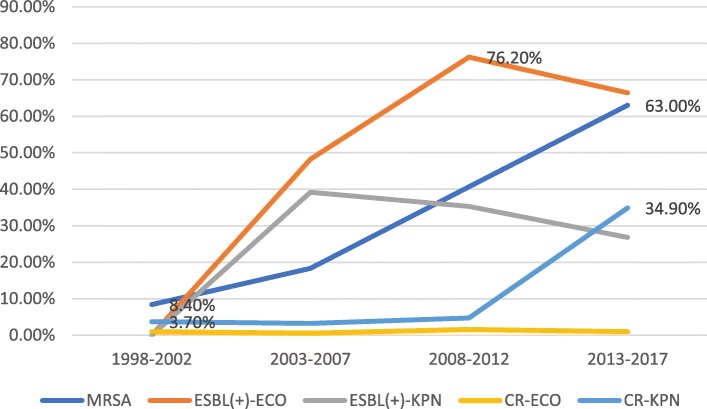
Distribution of multi drug resistant strains in hospitalized non ICU patients in 1998–2017

**Fig. 7:**
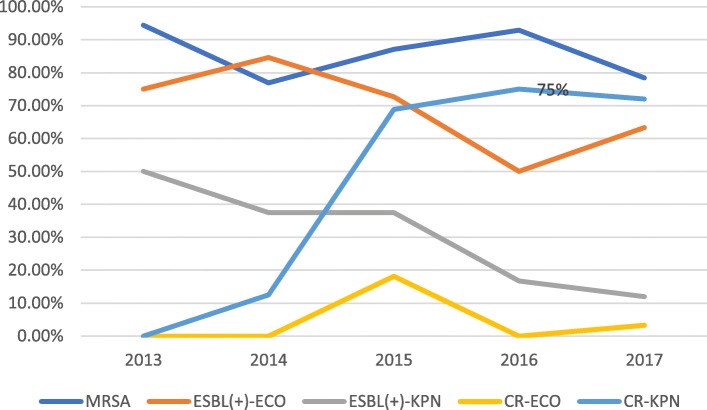
Distribution of multiple drug resistant strains in ICU patients in 2013–2017

The sensitivity rates for *S. aureus* to vancomycin, teicoplanin and trimethoprim/sulfamethoxazole were higher than 80%, while sensitivity to penicillin was lower than 10%. Sensitivity to oxacillin, gentamicin and levofloxacin in *Staphylococcus aureus* showed a significant downward trend. Erythromycin and clindamycin sensitivity in *Staphylococcus aureus* fluctuated slightly, ranging from 30 to 50% and from 50 to 60%, respectively (Fig. 4). The sensitivity rates for *K. pneumoniae* to imipenem, meropenem and amikacin were above 60%, and this pathogen was naturally resistant to ampicillin. The changing trend of the susceptibility rate, with the exception of trimethoprim/sulfamethoxazole showed that the susceptibility rates of the other antimicrobial agents in 2013–2017 were significantly lower than those in 2008–2012 (Fig. 5). According to the changing trend for the number of multidrug resistant strains of methicillin-resistant*Staphylococcus aureus* (MRSA), extended-spectrum beta-lactamase (ESBL)-producing *E. coli* and *K. pneumoniae*, carbapenem-resistant (CR) *E. coli* and *K. pneumoniae*, MRSA and CR *K. pneumoniae* all showed an upward trend (Fig. 6). The data for 2013 to 2017 showed that carbapenem-resistant*K. pneumoniae* in ICU patients was on the rise (Fig. 7).

## Discussion

The monitoring data for Tongji Hospital from 1998 to 2017 showed that the main pathogens causing BSIs were *E. coli* and *Staphylococcus aureus*, a finding consistent with previous reports from EARS-Net, Kor-GLASS and HBARSS [2, 3, 5], but inconsistent with reports from African countries where the main BSI-causing pathogens were non-typhoid*Salmonella* and *Salmonella typhi* [1, 10]. In our data, *S. typhimurium* was the third most common cause of BSIs during 1998–2002, after which it gradually decreased. *Salmonella* infections are food-borne diseases in which patients are mainly infected by eating *Salmonella*-contaminated food or drinking water [11]. *Salmonella* infections were severe in their clinical manifestations in African countries, and in our region from 1998 to 2002 also, which may be related to the health and medical conditions locally. Our study also found that CR *K. pneumoniae*, whether in ICU or non-ICU patients, significantly increased the incidence of BSIs. Data from EARS-Net showed that CR *K. pneumoniae* is a problem that should not be ignored in different European countries and in different age groups [12]. Infections caused by antibiotic-resistant bacteria such as MRSA, bacteria that carry ESBL genes or CR genes in *K. pneumoniae* and *E. coli*, threaten modern health care systems.

*E. coli* is a major cause of BSIs in Europe, and its rate of resistance to antibiotics had aroused critical concern throughout this continent [13–15]. From 1998 to 2017, we found that the susceptibility rates of *E. coli* to first-generation cefazolin, second-generation cefazolin and cefuroxime, third-generation cefazolin, cefotaxime and ceftazidime, fourth-generation cefazolin, cefepime and aztreonam decreased significantly in 2017 compared with 1998. Concurrently, we also found that the proportion of ESBLs produced by *E. coli* increased significantly, from 0% in 1998–2002 to 76.2% in 2008–2012. The rapidly increasing incidence of ESBL-expressing bacteria explains the high resistance rate of *E. coli* to cephalosporins and aztreonam. Whether ESBLs are associated with mortality has proved a controversial topic in different studies [16–20]. However, ESBL-producing strains can prolong hospitalization times, increase medical expenditure, and inflict higher financial burdens on patients with these infections [21, 22]. Studies have shown that the total length of hospitalization, the length of hospitalization before the infection, and the use of combined antibiotics and aminoglycosides are important risk factors for ESBL-related bloodstream infections [22].

We also found that *Staphylococcus aureus* was the most common causative agent of BSIs, and this bacterium was most sensitive to vancomycin and teicoplanin glycopeptide antibiotics with a sensitivity rate of 100% in 1998–2017. Trimethoprim and sulfamethoxazole were also the antibiotics that *Staphylococcus aureus* was most sensitive to, with a sensitivity rate above 80%. However, the sensitivity of *Staphylococcus aureus* to levofloxacin and gentamicin declined over time. The isolation rate of MRSA increased significantly from 8.4% in 1998–2002 to 68.3% in 2013–2017. The isolation rate of MRSA in the study region was significantly higher than that (36.6%) reported by multi-center monitoring of 22 teaching hospitals in 12 Chinese cities in 2016 [23]. Moreover, the Chinese multicentric monitoring data for 2013 to 2016 and the data for Germany in 2014 showed that the sensitivity of MRSA to quinolones was increasing, but our data showed a decline [23, 24]. The possible reasons underlying this finding are related to the extensive use of quinolones and aminoglycosides in our study region. The data for 22 Hong Kong hospitals showed that the 30-day mortality rate for patients with MRSA-induced BSIs was 32.39% [25], revealing that MRSA-related BSIs were a significant burden in Hong Kong [25], as they were also in our study area.

It has been found that after *E. coli*, *K. pneumoniae* is the predominant BSI-causing pathogen [26–28], and CR is the most serious problem in BSIs caused by it. In the present study, the detection rate for CR *K. pneumoniae* in non-ICU patients increased from 3.7% in 1998–2002 to 34.9% in 2013–2017. In ICU patients, the detection rate for CR *K. pneumoniae* increased from 0% in 2013 to 75% in 2016. Consistent with this finding, the surveillance data from CHINET in China showed that CR *K. pneumoniae* increased significantly from 2005 to 2014 [29]. Carbapenems have been used as the first-line antibiotics for treating multidrug-resistant*K. pneumoniae* [30]. However, resistance to carbapenems is making the treatment of CR *K. pneumoniae* extremely difficult. The results of a retrospective study conducted at a Shanghai teaching hospital revealed an overall 30-day mortality rate for BSIs of 25% for patients who had contracted CR *K. pneumoniae* [31]. Another retrospective cohort study on the effect of appropriate combination therapy on mortality in patients with BSIs caused by carbapenemase-producing*Enterobacteriaceae* reported that use of combination therapy was associated with improved survival only in patients with a high mortality risk score [32]. The authors also reported that patients with BSIs caused by carbapenemase-producing*Enterobacteriaceae* should receive active therapy upon diagnosis, and monotherapy should be considered for those in the low mortality-score stratum [32].

The present study is the first report on the accumulated data from our center at Tongji Hospital covering the past 20 years. Our findings augment current understanding on the pathogenic bacteria responsible for BSIs and the changes in drug resistance occurring in them over time in central China.

## Conclusions

The present focus of bacterial resistance control is to curb the spread of multi-drug resistant strains, especially CR *K. pneumoniae*, the infectious from which have increased significantly in recent years, thereby meriting special attention. Finally, controlling infections with MRSA remains an important goal.

## Data Availability

The datasets used and/or analyzed during the current study are available from the corresponding author on reasonable request.

## Abbreviations

BSIs: Bacterial bloodstream infections
CAP: College of American Pathologists
CLSI: Clinical & Laboratory Standards Institute
CR: carbapenem-resistant
EARS-Net: European Antimicrobial Resistance Surveillance Network
ESBL: extended-spectrum beta-lactamase
HBARSS: Hubei Province Antimicrobial Resistance Surveillance System
ICUs: intensive care units
MRSA: methicillin-resistant *Staphylococcus aureus*
WBC: white blood cell
